# Editorial: New approaches to how bilingualism shapes cognition and the brain across the lifespan: Beyond the false dichotomy of advantage versus no advantage

**DOI:** 10.3389/fpsyg.2023.1149062

**Published:** 2023-02-07

**Authors:** Mark Antoniou, Christos Pliatsikas, Scott R. Schroeder

**Affiliations:** ^1^The MARCS Institute for Brain, Behaviour and Development, Western Sydney University, Penrith, NSW, Australia; ^2^School of Psychology and Clinical Language Sciences, University of Reading, Reading, United Kingdom; ^3^Centro de Investigación Nebrija en Cognición, Universidad Nebrija, Madrid, Spain; ^4^Department of Speech-Language-Hearing Sciences, Hofstra University, Hempstead, NY, United States

**Keywords:** bilingualism, cognition, brain, advantage, new approaches

For much of the 20^th^ century, bilingualism was thought to result in cognitive disadvantages. In recent decades, however, research findings have suggested that experience with multiple languages may yield cognitive benefits and even counteract age-related cognitive decline, possibly delaying the manifestation of symptoms of dementia. Subsequently, conflicting evidence has emerged, and this has led to questions regarding the robustness and generalizability of these claims. A heated debate has raged for more than a decade (Antoniou, 2019), with certain research groups consistently finding support for a bilingual advantage, and others consistently finding none. The field has reached a stalemate, which has stifled research opportunities and the advancement of knowledge. In organizing the present Research Topic, we sought contributions describing new approaches needed to advance our field. These contributions help move the field beyond the traditional framing of bilingualism as a binary variable and toward approaches that capture the dynamic nature of effects relating to bilingualism and cognition.

## New conceptualizations

One way of moving beyond traditional framing is to explore new conceptualizations of bilingualism, itself, and the relationship between bilingualism and cognition.

In her opinion piece, Bialystok likens the bilingual advantage debate to COVID-19 debates concerning which public health measures and mandates should (or should not) be implemented. She quotes virologist, Ian Mackay, who applied Reason's (1990) Swiss cheese model to COVID-19 risk mitigation by proposing that individual measures are imperfect (containing holes like a slice of Swiss cheese) and that only a multi-layered approach has sufficient redundancy built in to successfully offer protection from the risks at hand (similar to stacking slices of Swiss cheese so that the holes become covered). By adopting this metaphor, Bialystok is proposing that our field should move beyond simple conceptions concerning the relationship between bilingualism and cognition. Through this lens, bilingualism offers a layer of cognitive protection, but one which is porous rather than absolute. Bialystok's framing serves as a reminder that we, as a field, need to move beyond the “all or nothing” framing that has featured throughout the bilingual advantage debate over the past two decades.

The contribution from Sanches de Oliveira and Bullock Oliveira argues that the question of whether there are bilingual advantages in cognition is ill-formed and unanswerable. Bilingualism is a problematic category, according to the authors, because bilingualism and monolingualism are on a continuum rather than discrete, and languages and dialects are likewise on a continuum; what is more, a person's language proficiency is variable and skill- and context-specific, and full proficiency in any language is not even attainable, as one cannot have full proficiency in the vocabulary jargon of every possible activity. Cognition (and by extension cognitive advantages) are similarly problematic concepts, Sanches de Oliveira and Bullock Oliveira claim, partly because such concepts fail to account for the context-specific and thus variable nature of cognitive functioning.

Wagner et al. explore the questions of what it means to be bilingual, and what people consider to be a language. In doing so, they address the concern that many studies rely on participants' judgments of whether they themselves belong in the bilingual group or monolingual group. This self-assignment can be problematic because participants might vary considerably in what they believe constitutes a bilingual and even a language. In a survey of 528 participants, Wagner et al. observe a range of responses from participants when judging whether fictional speakers qualified as bilingual and fictional linguistic systems qualified as a language. Participants' definitions of bilingualism depended on several factors, including continued use of a language after immigrating and the presence of a writing system. Participants' definitions of a language depended on the presence of a writing system, similarity to other languages, and geographic breadth. Wagner et al. conclude that the variable and potentially inaccurate conceptions of bilingualism and language could contribute to some of the variable findings in the literature.

Chung-Fat-Yim et al. discuss the nuanced nature of attention, dividing this multi-faceted concept into sustained attention, selective attention, alternating attention, divided attention, and disengagement of attention. For each component of attention, the authors review relevant models from the psychology and neuroscience literature, as well as empirical research that has examined bilingualism's potential positive effects.

Voits et al. discuss the commonalities and complementarities between the bilingualism and cognitive aging literatures. Bilingualism tends to be reduced to a dichotomous trait, which misrepresents its status as a complex experience; other times it is overlooked as a contributory factor all together. These authors discuss why bilingualism is not recognized as a contributor to cognitive reserve. They also helpfully suggest how bilingualism can be better integrated into aging research in future work. A model of aging is needed that encompasses the contributions of lifestyle factors, one of which is likely to be bilingual experience.

## New measures

Another way of moving beyond the stalemate debate surrounding bilingual benefits is to create new tasks, measures, and analyses.

Wu and Struys examine the influence of language dominance on bilingual word recognition. Uyghur-Chinese bilinguals completed lexical decision tasks administered in the L1 and L2, as well as a flanker task. Although bilinguals differed in their language dominance, all reported that they preferred reading in Chinese, their L2. Consequently, better performance was observed in their L2 than L1 on the lexical decision tasks. Further, those who had acquired their L2 earlier and had higher across-modality dominance in the L2 tended to recognize L2 words faster. The findings suggest that language dominance may be operationalized as a continuous or a categorical variable, and in doing so may exhibit effects not only for lexical recognition but also indirectly impacting domain-general contributions to recognition.

van den Berg et al. also investigate how individual bilingual experiences affect executive control in different contexts of language use. They tested bilingual university students, for whom they calculated a measure of language entropy for different contexts (university and non-university) by using a language background questionnaire. These language entropy measures were used as continuous predictors of the participants' performance in a color-shape switching task. Apart from collecting Reaction Times, pupil size was also measured as an objective index of set shifting abilities that are required for this task. The authors report that, while typical switching costs in RTs were not affected by entropy in either context, entropy did predict a switching cost in a non-university context when pupil dilation was studied. van den Berg et al. conclude that social diversity in bilinguals' experiences may indeed be linked to their executive control abilities, but this may depend on the exact social context and may be detectable in measures that are more sensitive than RT, such as pupil size.

Similarly, Freeman et al. focus on how quantified individual bilingual experiences affect performance in a non-linguistic task tapping executive control. Specifically, a sample of 146 Spanish-English heritage bilinguals were tested in a Stroop arrows task, from which the Stroop, facilitation and inhibition effects were calculated. Measures of individual experiences were used as predictors of these effects, including participants' sociolinguistic context (categorical), a composite continuous variable indexing L2 proficiency and exposure, as well as L2 age of acquisition, L2 proficiency and a measure of non-verbal cognitive reasoning, all continuous factors. The authors report a rich pattern of findings which converged in that increased bilingual experiences and cognitive skills led to increased abilities of focusing on relevant stimuli while ignoring irrelevant ones. These findings were also modulated by the sociolinguistic environment of the individuals, suggesting that any effects of bilingualism on cognition should be viewed in relation to the contexts that bilinguals find themselves in.

Grant et al.'s contribution follows on the same path of avoiding a binary monolingual-bilingual comparison and employing a seldom-used but meaningful and sensitive neural measure. Specifically, participants listened to speech-in-noise in their L1 and L2; the continuous independent variable of L2 age of acquisition and the dependent variable of EEG-measured alpha power were used. Findings indicate an increased alpha power when listening in the L2 and when the participant had an older L2 age of acquisition.

In a similar vein, Marin-Marin et al. turn their attention to the effects of bilingualism on brain structure, by using a measure of bilingual experiences as a predictor of regional gray matter volume in a group of Catalan-Spanish bilinguals that were immersed in a bilingual environment. They report non-linear volumetric fluctuations in a series of cortical and subcortical regions that have been linked to speech processing and language control. The authors argue that their pattern of results are corroborative of theoretical suggestions for dynamic, non-linear effects of bilingualism on the adult brain.

Finally, Dash et al. attempt to advance modeling bilingualism as a continuous variable. They show that a multifactorial approach to different dimensions of bilingual study may lead to a better understanding of the role of bilingualism on cognitive performance. Rather than reducing variability or treating it as problematic, these authors argue that variability needs to be embraced in bilingual profiles if we are to generalize the results of individual studies to the wider literature.

## Future directions

Taken together, the articles within this Research Topic provide suggestions concerning how our field might move beyond the entrenched positions that have characterized the bilingual advantage debate for more than a decade. We are excited by the ambitious and rigorous studies that will emerge in coming years to advance understanding of how experience with multiple languages interacts with other variables to affect cognition, the structure and function of the brain, and aging. There remains a need for detailed theoretical models that generate testable predictions in order for us to understand what types of bilingual experiences are more (or less) likely to show plasticity effects in a given domain. To achieve this, it is necessary to pay attention to how bilingualism is conceptualized and to methodological nuances in experimental designs, such as differences in tasks used and in the components of cognition they measure. By focusing on these aspects, we believe that this Research Topic offers a window into how knowledge can advance within our field, specifically concerning how bilingualism affects cognition and the brain.

## Author contributions

MA, CP, and SRS contributed equally to the writing of this editorial article. All authors contributed to the article and approved the submitted version.
