# Corrigendum: Editorial: New approaches to how bilingualism shapes cognition and the brain across the lifespan: beyond the false dichotomy of advantage versus no advantage

**DOI:** 10.3389/fpsyg.2023.1235628

**Published:** 2023-06-21

**Authors:** Mark Antoniou, Christos Pliatsikas, Scott R. Schroeder

**Affiliations:** ^1^The MARCS Institute for Brain, Behaviour and Development, Western Sydney University, Penrith, NSW, Australia; ^2^School of Psychology and Clinical Language Sciences, University of Reading, Reading, United Kingdom; ^3^Centro de Investigación Nebrija en Cognición, Universidad Nebrija, Madrid, Spain; ^4^Department of Speech-Language-Hearing Sciences, Hofstra University, Hempstead, NY, United States

**Keywords:** bilingualism, cognition, brain, advantage, new approaches

In the published article, there was an error. The summary of one of the papers included in the Research Topic to which this Editorial relates erroneously stated that two groups were tested in different contexts.

A correction has been made to Section **New measures**, paragraph 3. The corrected section is shown below.

“van den Berg et al. also investigate how individual bilingual experiences affect executive control in different contexts of language use. They tested bilingual university students, for whom they calculated a measure of language entropy for different contexts (university and non-university) by using a language background questionnaire. These language entropy measures were used as continuous predictors of the participants' performance in a color-shape switching task. Apart from collecting Reaction Times, pupil size was also measured as an objective index of set shifting abilities that are required for this task. The authors report that, while typical switching costs in RTs were not affected by entropy in either context, entropy did predict a switching cost in a non-university context when pupil dilation was studied. van den Berg et al. conclude that social diversity in bilinguals' experiences may indeed be linked to their executive control abilities, but this may depend on the exact social context and may be detectable in measures that are more sensitive than RT, such as pupil size.”

The authors apologize for this error and state that this does not change the scientific conclusions of the article in any way. The original article has been updated.

